# Immunomodulation and Protective Effects of *Cordyceps militaris* Extract Against *Candida albicans* Infection in *Galleria mellonella* Larvae

**DOI:** 10.3390/insects15110882

**Published:** 2024-11-10

**Authors:** Kiratiya Eiamthaworn, David Holthaus, Sureeporn Suriyaprom, Volker Rickerts, Yingmanee Tragoolpua

**Affiliations:** 1 Department of Biology, Faculty of Science, Chiang Mai University, Chiang Mai 50200, Thailand; kirati.eiam@hotmail.com (K.E.); sureeporn.suriyaprom@cmu.ac.th (S.S.); 2 Department of Gynecology and Obstetrics, Universitätsklinikum Schleswig-Holstein, 24105 Kiel, Germany; david.holthaus@uksh.de; 3 Robert Koch Institute, 13353 Berlin, Germany; rickertsv@rki.de; 4 Office of Research Administration, Chiang Mai University, Chiang Mai 50200, Thailand; 5 Natural Extracts and Innovative Products for Alternative Healthcare Research Group, Chiang Mai University, Chiang Mai 50200, Thailand

**Keywords:** *Candida albicans*, *Cordyceps militaris* extract, *Galleria mellonella*, antimicrobial peptides (AMPs), immune response, infection models

## Abstract

*Cordyceps militaris*-derived formulations are currently used for multiple purposes because of their medical properties, especially immune system modulation. This study analyzes the inhibitory effects of *C. militaris* aqueous extract on *Candida albicans* infections and the immune response in larvae of the greater wax moth *Galleria mellonella* (Lepidoptera: Pyralidae). Larvae exhibited melanization within 1 h of being infected with *C. albicans* inoculum at a concentration of 10^6^ cells/larvae, and died within 24 h from a lethal dose. Aqueous extract of *C. militaris* proved to be nontoxic at concentrations of 0.25 and 0.125 mg/larvae, and had the greatest ability to prolong the survival of larvae infected with a sublethal dose of *C. albicans* at a concentration of 10^5^ cells/larvae. Moreover, the number of hemocytes in the hemolymph of *G. mellonella* increased after infection with *C. albicans* and treatment with the aqueous extract of *C. militaris* at 1, 24, and 48 h by 1.21 × 10^7^, 1.23 × 10^7^, and 1.4 × 10^7^ cells/100 µL, respectively. The highest number of hemocytes was recorded after treatment of infected *G. mellonella* with the extract for 48 h. Transcriptional upregulation of the immune system was observed in certain antimicrobial peptides (AMPs), showing that the relative expression of *galiomicin*, *gallerimycin*, and *lysozyme* genes were upregulated as early as 1 h after infection. Therefore, we conclude that *C. militaris* aqueous extract can modulate the immune system of *G. mellonella* and protect against infection from *C. albicans*.

## Simple Summary

*Candida albicans* can cause common mucosal and severe systemic infections, especially in immunocompromised patients. Current usage of antifungal drugs often leads to side effects and the development of drug-resistant strains. *Cordyceps militaris*, a parasitic fungus with various medicinal properties, is known for its immunomodulatory role. This study addresses the need for effective treatments against *C. albicans* infections by analyzing the inhibitory effects of *C. militaris* aqueous extract on *C. albicans* and its impact on the immune response in larvae of the greater wax moth *Galleria mellonella*. *C. militaris* extract, at nontoxic concentrations, significantly prolonged the survival of larvae infected with a sublethal dose of *C. albicans*. Additionally, the extract boosted the immune response by increasing hemocyte count and upregulating genes responsible for producing antimicrobial peptides. These findings suggest that *C. militaris* extract can effectively modulate the immune system and provide protection against fungal infections. This research highlights the potential of *C. militaris* as a natural, safe, and effective treatment option for *C. albicans* infections. Therefore, the aqueous extract of *C. militaris* is contributing to the development of alternative medicines. In addition, the utilization of *G. mellonella* larvae in this study can reduce the reliance on mammalian models in medical research, benefiting both human health and scientific research.

## 1. Introduction

The *Cordyceps* genus is an entomogenous fungus that can infect pupae and larvae of lepidopteron insects [1,2]. It is a well-known ascomycete parasitic fungus [3] and is composed of more than 750 species that are found in Asia, Europe, and North America. *C. militaris* has been used for multiple purposes due to its medicinal properties known for curing various diseases [3] and can also modulate the immune system [4]. Studies on the use of *C. militaris* for pharmaceutical purposes are shifting to incorporate natural treatments, further fueling the green pharmacy revolution and creating safe and affordable treatment options [5]. The aqueous extract of *C. militaris* comprises several bioactive substances, such as cordycepin (3′-deoxyadenosine), polysaccharides, ergothioneine, and γ-aminobutyric acid (GABA). These chemicals confer a broad spectrum of therapeutic activities, including anti-inflammation, immunomodulation, antioxidant, and anticancer activities. According to previous research, the aqueous extracts of *C. militaris* can stimulate macrophages to release immune cytokines such as IFN-γ, which is important for immune system modulation and may have therapeutic application for treatment of cancer and inflammation [4,6]. Various biological compounds have been found in *C. militaris*, such as γ-aminobutyric acid (GABA), cordycepin, lovastatin, ergothioneine, cordycepic acid, xanthophylls, carotenoids, ergosterol, and phenolic compounds, e.g., phenolic acids and flavonoids, vitamins, and trace elements, i.e., magnesium, potassium, selenium, and sulfur [7,8,9].

Larvae of the greater wax moth *Galleria mellonella* (Lepidoptera: Pyralidae) have gained popularity as infection models due to the functional similarities between their immune response after infection and the innate mammalian immune response [10,11,12,13]. *G. mellonella* can be used to assess the virulence of microbial pathogens that are comparable to those obtained from mammalian systems [14,15]. The innate immunity of *G. mellonella* exhibits similarities to mammalian innate immunity due to the presence of a cellular component [10,16,17]. Hemolymph compares favorably with mammalian blood as a dynamic tissue and contains immune cells known as hemocytes [17]. Hemocytes in *G. mellonella* play a role similar to mammalian macrophages and neutrophils, contributing to phagocytosis and clearance of pathogen after infection [18]. Moreover, *G. mellonella* produces antimicrobial peptides (AMPs) that are akin to those found in mammals, contributing to its capacity to fight infections. These factors, along with its ability to thrive at 37 °C [19], make it a valuable model for studying pathogen–host interactions and virulence mechanisms.

The fat body is the storage site for glycogen, lipids, and proteins, and is essential to metabolize energy. Thus, the fat body contributes to the preservation and mobilization of nutrients as well as the synthesis of proteins. These functions of the fat body are similar to the adipose tissues and the liver in vertebrates. Fat bodies are also the origin of immunological responses to pathogens, hematopoiesis, and several other immune components, such as antibacterial compounds and blood-clotting proteins [20].

At present, the use of *G. mellonella* is expanding, since this model could replace vertebrate infection models in order to reduce the number of mammals used in research [21]. The larvae of the greater wax moth, *G. mellonella*, are well accepted as a model for the assessment of microbial pathogen virulence factors and the effectiveness of antimicrobial agents after microbial infection and treatment [22,23]. These larvae have demonstrated the ability to be a proper host of fungi to evaluate the effects of antifungal drugs and new compounds with suspected antifungal properties [24]. Other advantages of the *G. mellonella* model are the cost efficiency and the simplicity in handling. Additionally, *G. mellonella* can be cultured at 37 °C without requiring any adaptations to the laboratory. These factors have resulted in an increase in the use of these larvae to study fungal virulence, antifungal drug activity, and alternative medicine [25].

*Candida albicans* is one of many fungi that causes skin disease that persistently colonizes and invades host tissues. It is also a predominant opportunistic fungal pathogen, leading to cutaneous candidiasis disease [26]. Cutaneous candidiasis can be treated with antifungals and steroid therapy. Antibiotics are an important part of contemporary treatments; however, they are becoming ineffective due to the development of microbial resistance [27]. At least 700,000 people per year, and potentially 10 million people by 2050, are expected to suffer from drug-resistant microorganisms [27]. Thus, drug development from natural substances provides viable alternative therapies for treating microbial infections and inflammation. Natural drugs are considered to be less toxic than synthetic ones, and have few side effects. Nowadays, natural compounds or secondary metabolites from fungi are gaining interest and considered to be antimicrobial agents. As important medicinal fungi, *C. militaris* has also been tested for its antimicrobial activities [28]. However, its biological activities of *C. militaris* extract against *C. albicans,* the stimulation of the immune response in *G. mellonella* after *C. albicans* infection, and treatment with the aqueous extract of *C. militaris* have never been investigated [29]. Therefore, the inhibitory effects of the *C. militaris* aqueous extract on *C. albicans* and the immune response in *G. mellonella* after injection of *C. albicans* into *G. mellonella* were determined.

## 2. Materials and Methods

### 2.1. Preparation of C. militaris Extract, G. mellonella, and C. albicans

*C*. *militaris* fruiting body was obtained from the Mushroom Research and Development Center, Chiang Mai Province, Thailand. The dried fruiting body was ground into a fine powder. The powder was then soaked in sterile, distilled water at a ratio of 100 g of *C*. *militaris* powder to 1 L of solvent, kept at 45 °C for 3 h, and shaken every 30 min. The extraction was performed twice. Thereafter, the extract was filtered through filter paper No. 1 (Whatman™, Buckinghamshire, UK), evaporated, and lyophilized (LABCONCO, Kansas City, MO, USA) to obtain a crude extract. The crude extract was dissolved in sterile water before use [30]. The percentage yield was calculated from the dry weight as follows:

$$Percentage\;yield\;of\;crude\;extract\left(\%\right)=\frac{Weightofextract}{Weightofdry\;C.\;militaris}\times 100$$

In this study, we used the sixth instar larvae (late state) with light-color and the average weight of 263.02 ± 5.08 g without any grey color marker on the cuticle. The weight of *G. mellonella* larvae used in previous studies typically varies from 230–350 mg depending on their developmental stage, age, and environmental conditions [31]. Twenty larvae were used per each test group. During the experiment, *G. mellonella* were maintained in Petri dishes containing wood chips at 37 °C in dark conditions [32].

*C. albicans* SC5314 was grown in 50 mL of yeast peptone dextrose (YPD) broth (Thermo Scientific™, Waltham, MA, USA) at 37 °C and stimulated by shaking at 150 rpm for 15–17 h. The *C. albicans* cell pellet was harvested by centrifugation at 3000× *g* at 4 °C for 5 min. The pellets containing the *C. albicans* cells were then washed three times with 10 mL of 0.85% NaCl solution in sterile distilled water. The inoculum of *C. albicans* was prepared at 10^8^ cells/mL in 0.85% NaCl and injected into *G. mellonella* at 5 × 10^5^ cells/10 µL.

### 2.2. Determination of Toxicity of C. militaris Extract on G. mellonella

The aqueous extract of *C. militaris* was resuspended in sterile distilled water and filtered through a 0.22 µm Minisart^®^ NML cellulose acetate syringe filter (Sartorius, Göttingen, Germany) to prevent contamination from other microorganisms. Then, the extract of *C*. *militaris* was evaluated for toxicity through injection at a volume of 10 µL/larvae on *G. mellonella* using an Omnican^®^ 0.5 mL insulin syringe U-100 (B. Braun, Melsungen, Germany) for injection, and the survival rate was determined [32].

Before injection of *C*. *militaris* extract, larvae were cleaned by swabbing the last proleg region with 70% ethanol. To avoid leaking of hemolymph, the hemolymph was gently removed from the larvae at the last proleg region (the final pair of legs) using a 30-gauge needle. The toxicity was evaluated after 5 doses of injections (0.125, 0.25, 0.5, 1, and 2 mg/larvae, respectively) into each group of larvae and was compared to the control group that did not receive any injections. The larval mortality rate was recorded daily. If an extract did not exhibit toxicity, which is defined as having a larval survival rate above 50% in a group, it was classified as nontoxic and accepted for future investigation.

### 2.3. Determination of G. mellonella Immune Response After C. albicans Infection

The immune response of *G. mellonella* after *C*. *albicans* infection was determined in 6 groups following injections of *C*. *albicans* at concentrations of 10^4^, 10^5^, and 10^6^ cells/larvae. The control groups consisted of heat-inactivated (H/I; 65 °C for 30 min) 10^6^ cells/larvae, 0.85% NaCl, and no treatment, respectively. The heat-inactivated *C*. *albicans* was verified by plating of 5 µL of the pathogen on yeast peptone dextrose (YPD) agar (Thermo Scientific™, Waltham, MA, USA) to determine the colony of the inactivated pathogen [33,34].

*G. mellonella* larvae were weighed 24 h before the experiment and then incubated at 37 °C. The groups of *G. mellonella* larvae were cleaned with 70% ethanol to prepare for injection. Then, 10 µL of *C*. *albicans* was injected into larvae at the last left proleg using an insulin syringe. The larvae were then incubated at 37 °C for 30 min [35,36]. Thereafter, 10 µL of *C*. *militaris* extract was inoculated into the larvae on the opposite side of the first inoculation. The infected *G. mellonella* was transferred to a Petri dish containing wood chips and incubated at 37 °C for the duration of the experiment [37]. The health index was recorded as a reference score using the health index score system of *G. mellonella* larvae [38]. The health index is categorized as follows: unhealthy (0), partially healthy (0.5), healthy (1), and dead larvae (-). Moreover, the response of *G. mellonella* to *C. albicans* infection was assessed by observing cocoon formation and melanization. Cocoon production was classified into three levels: (none) larvae without cocoon, (partial) larvae with partial cocoon, and (full) larvae with full cocoon. Melanization was classified into four levels: (0) black larvae, (1) larvae with black spots on a brown background, (2) larvae exhibiting more than three spots, (3) larvae with fewer than three spots, and (4) control larvae without melanization.

### 2.4. Hemolymph and Fat Body Collection

*G. mellonella* larvae were infected with *C. albicans* and treated with *C. militaris* extract after 1, 24, and 48 h. Three infected *G. mellonella* larvae were cleaned by swabbing the last proleg region (the final pair of legs) with 70% ethanol and cut with sterile scissors from the lower part of the body to the top while the larvae were still alive without anesthesia. This last proleg region was chosen to minimize damage and provide access to the hemolymph while reducing the risk of gut infection. The hemolymph from three larvae was carefully aspirated into the syringe using a 30-gauge needle and kept in sterile microcentrifuge tube.

The hemolymph (30 µL/larvae) was added to 10 µL phenylthiourea (5 µg/mL) to prevent melanization. The hemocytes were then counted directly after collection using an improved Neubauer hemocytometer (Marienfeld, Lauda-Königshofen, Germany) under a microscope. After removal of the whole hemolymph, fat body from three larvae was collected at 1 h, 24 h, and 48 h in a collection tube. The fat body was collected after the larvae were surface sterilized with 70% ethanol. An incision was performed along the ventral midline, revealing the internal organs. The adipose tissue, a white membrane lining the body cavity, was meticulously excised from each larvae with forceps and collected in a 1.5 mL tube (Eppendorf, Hamburg, Germany) and resuspended in 700 µL of TRI reagent^®^ (Invitrogen, Waltham, MA, USA) for total RNA extraction, which was performed following the manufacturer’s guidelines. RNA concentrations and purity were determined by measuring the absorbance ratio at 260/280 nm using Infinite^®^ M200 PRO plate reader (Tecan, Männedorf, Switzerland) before gene expression analysis [32]. The experiment was performed in triplicate.

### 2.5. The Expression of Antimicrobial Peptide Genes of G. mellonella by Quantitative Reverse Transcription Polymerase Chain Reaction (qRT-PCR)

*G. mellonella* larvae were infected by *C*. *albicans* and treated with *C*. *militaris* extract as described above. Larval RNA was extracted from the fat body using a Direct-zol-RNA-Microprep kit (Zymogen, R2063, Zymo Research, Irvine, CA, USA) at 1, 24, and 48 h post-infection. Quantity of RNA was measured by an Infinite M200 Pro plate reader (Tecan, Männedorf, Switzerland) at 260/280 nm. To reverse-transcribe the RNA, the High-Capacity RNA-to-cDNA Kit (Applied Biosystems, Waltham, MA, USA) was used with 500 ng per reaction. Quantitative RT-PCR was performed using a system composed of a C1000 cycler and CFX96 head (BioRad, Hercules, CA, USA). The program included an initial 10 min enzyme activation step at 95 °C, followed by 40 cycles of 20 s at 95 °C, 30 s at 60 °C and 20 s at 72 °C. Amplicon specificity was confirmed through melting curve analysis [39]. Relative expression levels of the selected AMPs *galiomicin*, *gallerimycin*, and *lysozyme* (Table 1) were quantified using the ΔΔ*C*_T_ method [40,41], using *G. mellonella β-actin* as the internal reference gene for RNA loading. To validate the method, the difference between the *C*_T_ values of each *AMPs* and *β-actin* [Δ*C*_T_ = *C*_T_ (*galiomicin*, *gallerimycin*, or *lysozyme*)  −  *C*_T_ (*β-actin*)] was plotted against the log of 10-fold serial dilutions (100, 10, and 1 ng/µL) of purified RNA samples. The plot of log total RNA input versus Δ*C*_T_ showed a slope of less than 0.1, indicating that the amplification efficiencies of the two amplicons were approximately equal.

### 2.6. Statistical Analysis

Statistical analyses were performed using GraphPad Prism 9.5.1 (GraphPad Software, Inc., San Diego, CA, USA). The survival curve was analyzed using the log-rank (Mantel–Cox) test, with a *p* value of ≤0.05 considered significant. For the statistical analysis of hemocyte count and antimicrobial peptide gene expression, two-way ANOVA (Tukey’s multiple comparison test) was employed. Normality of the data was assessed using the Shapiro–Wilk test, and homoscedasticity was evaluated with Levene’s test to ensure the validity of the ANOVA assumptions.

## 3. Results

### 3.1. The Fruiting Body of C. militaris Extraction

**Figure 1:**
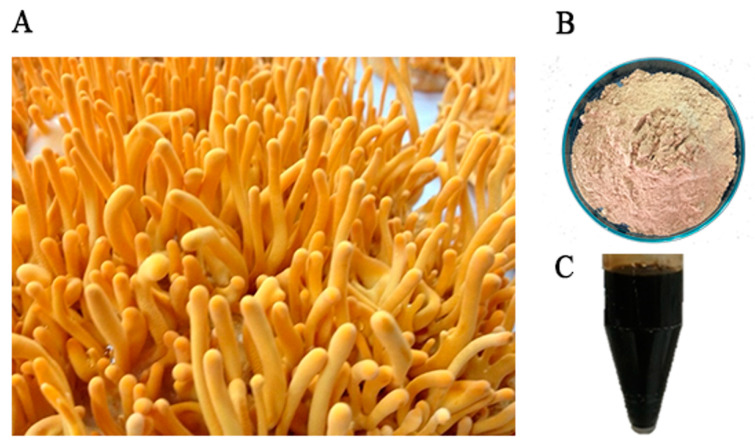
The extraction of *C. militaris* fruiting body. (**A**) The fruiting body of *C. militaris*, (**B**) the dried crude extract, and (**C**) the aqueous extract after dissolving in sterile distilled water.

The fruiting body of *C. militaris* was extracted with distilled water, resulting in a brown color of the dried crude extract with the percentage yield of 16.8% and a dark brown color of the aqueous extract after dissolving in sterile distilled water (Figure 1).

### 3.2. Determination of G. mellonella Survival After Treatment with C. militaris Extract 

**Figure 2:**
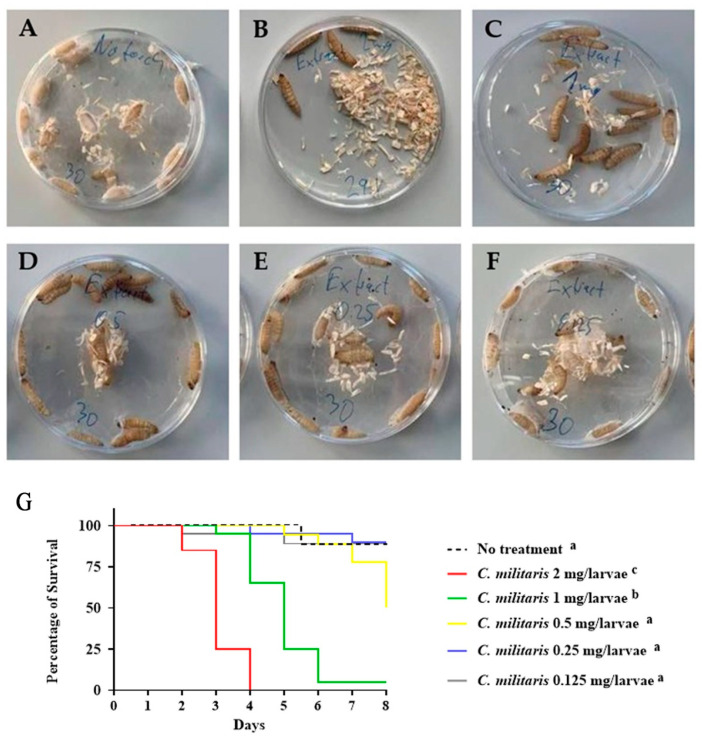
Survival of *G. mellonella* larvae after treatment with different concentrations of *C. militaris* extract. Survival rates were determined using the Kaplan–Meier test. (**A**) No treatment (control), (**B**) 2 mg/larvae, (**C**) 1 mg/larvae, (**D**) 0.50 mg/larvae, (**E**) 0.25 mg/larvae, (**F**) 0.125 mg/larvae, and (**G**) toxicity of *C. militaris* extract on *G. mellonella* at 0.125 (grey line), 0.25 (blue line), 0.5 (yellow line), 1 (green line), and 2 mg/larvae (red line). Significant difference was shown when treating with *C. militaris* extracts at 1 and 2 mg/larvae using log-rank test (χ^2^ = 128.1, dF = 5, *n* = 20, and *p* < 0.0001), and no significant difference was observed between “no-treatment” control and the group treated with *C. militaris* extracts at 0.125, 0.25, and 0.5 mg/larvae. Kaplan–Meier survival curves were determined from three independent experiments. The superscript letters (a–c) represent statistically significant differences between the Kaplan–Meier survival curves. Curves designated with the same letter are not significantly different from one another.

After treating *G. mellonella* larvae with *C*. *militaris* extract at a concentration of 2 mg/larvae group, the larvae moved slower on day 2 and all larvae died on day 4. By this time, some of the dead larvae turned to a red–brown color on the cuticle (Figure 2 and Tables S1 and S2). The extract of *C*. *militaris* at concentrations of 0.25 and 0.125 mg/larvae was able to prevent larvae from forming pupae and producing a cocoon. Importantly, these indicated doses did not exhibit lethal effects compared to the control group, thereby allowing the larvae to remain viable. Additionally, the extract did not have a toxic effect on the larvae, and they remained alive longer than the larvae in other groups until the end of the experiment (log-rank test: *p* < 0.0001). Based on these results, *C*. *militaris* extract at concentrations of 0.25 and 0.125 mg/larvae were selected to study the effects of *C*. *militaris* extract on experimental *G. mellonella* larval candidiasis.

### 3.3. Determination of G. mellonella Survival After C. albicans Infection

**Figure 3:**
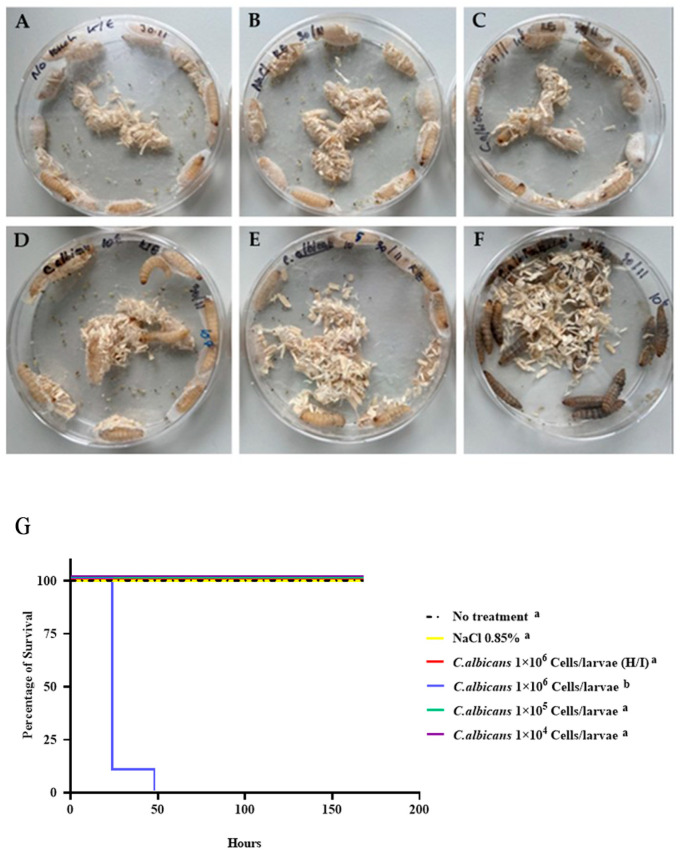
Survival of *G. mellonella* larvae after *C. albicans* infection. (**A**) No treatment (control), (**B**) 0.85% NaCl, (**C**) *C. albicans* heat-inactivated (H/I), (**D**) *C. albicans* 10^4^ cells/larvae, (**E**) *C. albicans* 10^5^ cells/larvae, (**F**) *C. albicans* 10^6^ cells/larvae, and (**G**) survival of *G. mellonella* after infection with *C. albicans* at 10^4^ cells/larvae (purple line), 10^5^ cells/larvae (green line), 10^6^ cells/larvae (blue line), 10^6^ heat-inactivated (H/I) cells/larvae (red line), and 0.85% NaCl (yellow line) compared with no treatment (control). There was no significant difference in each group of samples, except 10^6^ cells/larvae, which exhibited a highly significant difference using log-rank test (χ^2^ = 110.5, dF = 5, *n* = 20, and *p* < 0.0001) compared with no treatment (control). Kaplan–Meier survival curves were determined from three independent experiments. The superscript letters (a,b) represent statistically significant differences between the Kaplan–Meier survival curves. Curves designated with the same letter are not significantly different from one another.

The immune response of *G. mellonella* after infection with *C*. *albicans* was observed in six groups, including 10^4^, 10^5^, and 10^6^ cells/larvae, *C*. *albicans* heat-inactivated (H/I) 10^6^ cells/larvae, 0.85% NaCl, and the no-treatment (control) group, respectively. When *C. albicans* inoculum was injected into the proleg of *G. mellonella* at a concentration of 10^6^ cells/larvae, the larvae expressed melanization within 1 h after being infected and died within 24 h by 90% and 100% within 48 h (Figure 3 and Tables S3 and S4). As the inoculum of 10^6^ cells/larvae was found to be unsuitable for evaluating the immune response of the *G. mellonella* larvae, *C*. *albicans* at a concentration of 10^5^ cells/larvae was selected for further study.

### 3.4. Response of G. mellonella After C. albicans Infection by Determination of Cocoon Formation and Melanization

**Figure 4:**
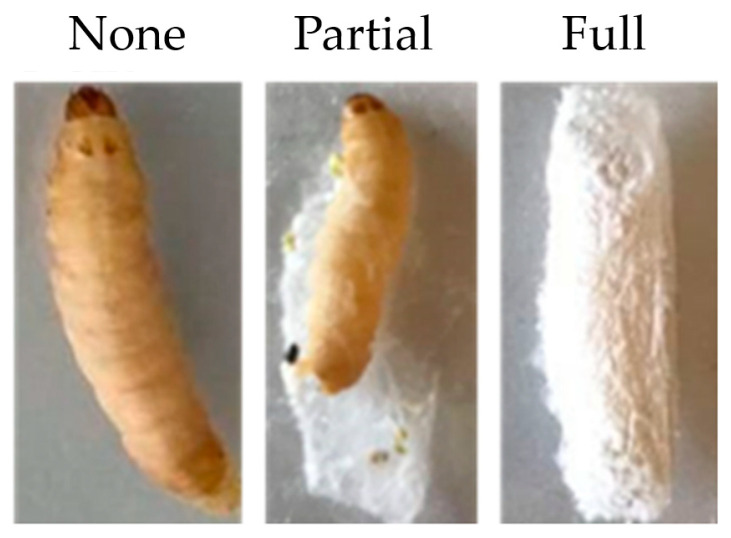
Cocoon production of *G. mellonella* after *C. albicans* infection for more than 24 h. None is larvae without cocoon, partial is larvae with partial cocoon, and full is larvae with full cocoon.

Cocoon production was determined after *G. mellonella* was infected with *C. albicans* at concentrations of 10^4^, 10^5^, 10^6^ cells/larvae, *C. albicans* heat-inactivated (H/I) 10^6^ cells/larvae, 0.85% NaCl, and no-treatment (control) groups (Table 2). The results showed that each group of *G. mellonella* still produced a cocoon, except for the larvae group that was infected with *C. albicans* at 10^6^ cells/larvae, which could not produce a cocoon after injection. During the observation period and until the end of the experiment, *G. mellonella* could produce some part of the cocoon after infection with *C*. *albicans* at 10^5^ cells/larvae (Figure 4). Thus, the cocoon formation correlated with the survival result.

**Figure 5:**
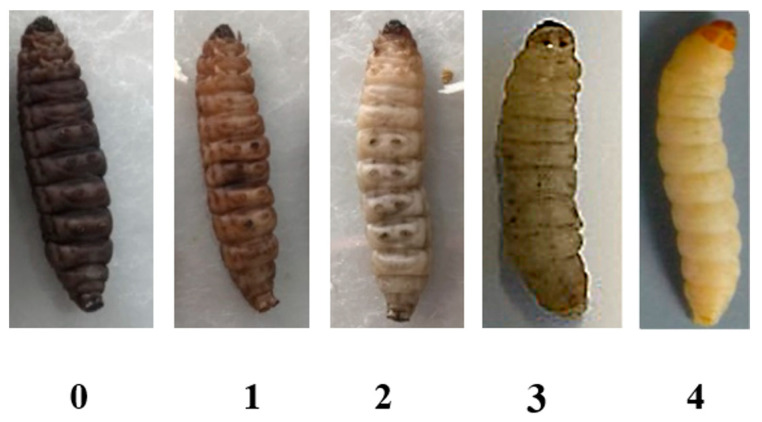
The level of melanization in *G. mellonella*: (**0**) black larvae, (**1**) black spots on brown larvae, (**2**) >3 spots on being larvae, (**3**) <3 spots on larvae, and (**4**) control without melanization.

**Figure 6:**
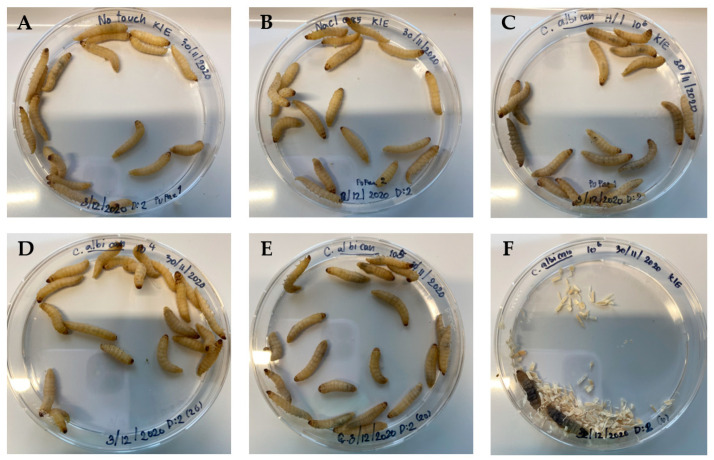
The melanization of *G. mellonella* after 24 h of *C*. *albicans* infection. (**A**) No treatment (control), (**B**) 0.85% NaCl, (**C**) *C*. *albicans* heat-inactivated (H/I), (**D**) *C*. *albicans* 10^4^ cells/larvae, (**E**) *C*. *albicans* 10^5^ cells/larvae, (**F**) *C*. *albicans* 10^6^ cells/larvae.

Another observation of *G. mellonella* at 24 h after *C. albicans* infection was the melanization of the larvae. The results exhibited that *G. mellonella* infected with 10^4^ cells of the *C. albicans*/larvae, 0.85% NaCl, and no-treatment (control) group did not show any melanization on *G. mellonella* cuticle (melanization level of 4), whereas *G. mellonella* infected with *C. albicans* at 10^5^ cells/larvae showed a melanization level of 3 (<3 spots on larvae). Moreover, *G. mellonella* infected with *C. albicans* heat-inactivated (H/I) exhibited black spots on larvae cuticle, caused by a melanization level of 2 (>3 spots on being larvae). Furthermore, *G. mellonella* infected with *C. albicans* at 10^6^ cells/larvae showed a melanization level of 1 and completely black color caused by a melanization level of 0, and the larvae rapidly died (Figure 5 and Figure 6).

### 3.5. The Therapeutic Effects of C. militaris Extract in G. mellonella Infected with C. albicans

**Figure 7:**
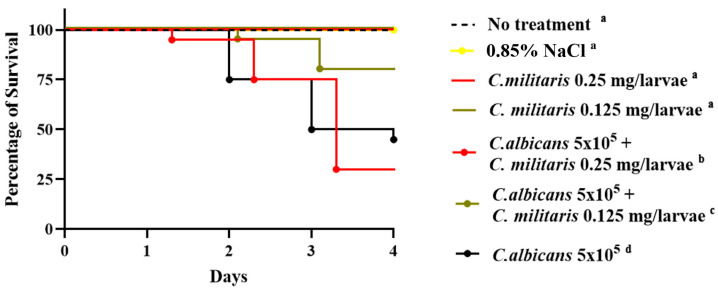
Survival of *G. mellonella* after infection with *C*. *albicans* at 5 × 10^5^ cells/larvae and treated with *C*. *militaris* extract at 0.125 and 0.25 mg/larvae. The survival of *G. mellonella* after infection with *C*. *albicans* at 5 × 10^5^ cells/larvae was found to be significantly different between the *C*. *militaris* treatment groups (0.125 mg/larvae and 0.25 mg/larvae using log-rank test (χ^2^ = 58.46, dF = 5, *n* = 20, and *p* < 0.0001)) when compared to the *C*. *albicans*-infected *G. mellonella* control group. There was no significant difference in the survival of *G. mellonella* after treatment with 0.85% NaCl or *C. militaris* extract at 0.125 and 0.25 mg/larvae compared to the no-treatment (control) group. The superscript letters (a–d) represent statistically significant differences between the Kaplan–Meier survival curves. Curves designated with the same letter are not significantly different from one another.

*C*. *militaris* extracts at concentrations of 0.125 and 0.25 mg/larvae, which proved to be nontoxic to the survival of larvae, were selected for study. When *G. mellonella* were injected with *C*. *albicans* at a concentration of 5 × 10^5^ cells/larvae and treated with *C*. *militaris* extract at a concentration of 0.25 mg/larvae, the larvae exhibited a higher mortality compared to the group infected with only *C*. *albicans*. Therefore, *C*. *militaris* at a concentration of 0.25 mg/larvae did not show any therapeutic effects on *G. mellonella*. However, when treated with *C. militaris* extract at a concentration of 0.125 mg/larvae, *G. mellonella* larvae exhibited a longer survival time compared to the group infected with *C. albicans* alone (log-rank test: *p* < 0.0001). Consequently, the concentration of *C*. *militaris* extract at 0.125 mg/larvae was chosen for further investigation of antimicrobial peptide gene expression (Figure 7 and Tables S5 and S6).

### 3.6. Determination of Hemocytes After Infection from C. albicans

**Figure 8:**
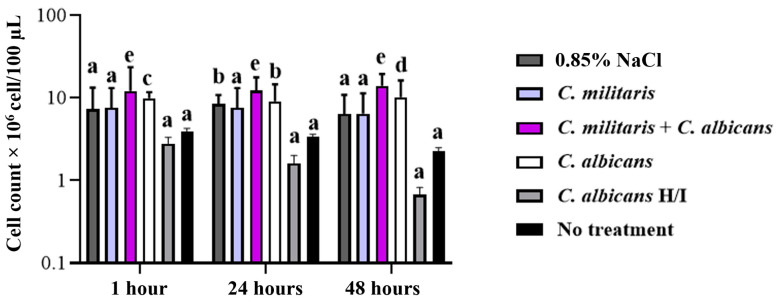
The increased hemocytes of *G. mellonella* after *C. albicans* infection at 5 × 10^5^ cells/larvae and treated with *C. militaris* extract at 0.125 mg/larvae for 1, 24, and 48 h using two-way ANOVA (Tukey’s multiple comparison test). ^a, b, c, d, e^ indicate a significant difference (*p* < 0.05) compared with the no-treatment group as the untreated cell control. The differences in hemocyte count are significantly influenced by treatment (two-way ANOVA: F(5, 36) = 40.83, dF = 5, *n* = 3, *p* < 0.0001). Time does not have a significant impact on hemocyte count (two-way ANOVA: F(2, 36) = 0.4433, dF = 2, *n* = 3, *p* = 0.6454), and there is no significant interaction between treatment and time (two-way ANOVA: F(10, 36) = 0.6714, dF = 10, *n* = 3, *p* = 0.7429).

To investigate the immune mechanisms associated with the preventive effects of *C*. *militaris* extract against *C*. *albicans* infection, the quantity of available hemocytes in the hemolymph of larvae after 1, 24, and 48 h of infection was studied (Figure 8 and Table S7). The hemolymph was collected by cutting the abdomen of the larvae. After *G. mellonella* was infected by *C. albicans* and treated with *C. militaris* extract for 1 and 24 h, a similar number of hemocytes was observed at 1.21 × 10^7^ and 1.23 × 10^7^ cells/100 µL, and the hemocyte count was greater than the no-treatment group, defined as an uninfected control group. Interestingly, the hemocytes of *G. mellonella* after infection by *C. albicans* and treated with *C. militaris* extract for 48 h were slightly greater at 1.40 × 10^7^ cells/100 µL. Thus, the hemocytes of *G. mellonella* after *C. albicans* infection and treatment with the extract for 1, 24, and 48 h were significantly higher than other groups, indicating that treatment plays a major role in determining hemocyte levels (two-way ANOVA: *p* < 0.0001). Interestingly, while treatment significantly influenced hemocyte count, time did not have a significant impact (*p* = 0.6454), and there was no significant interaction between treatment and time (*p* = 0.7429), suggesting that the treatment effect remains stable over time. These results suggest that *C. militaris* extract not only elevates hemocyte levels but also supports the immune response in *G. mellonella* during *C. albicans* infection.

### 3.7. Determination of G. mellonella Immune Response After C. albicans Infection and Treatment with C. militaris Extract

#### 3.7.1. Determination of *Galiomicin* Gene Expression in the *G. mellonella* Model

The upregulation of the immune system is demonstrated in *G. mellonella* during an immunological response to a pathogen. Monitoring the gene expression reveals certain antimicrobial peptides (AMPs). In this study, we aimed to quantify the expression of gene-encoding peptides implicated in immunological responses within the larvae. The immune response of *G. mellonella* in relation to the activation of the *galiomicin* gene, a defensin found in *G. mellonella*, was observed by quantitative reverse transcription polymerase chain reaction (qRT-PCR) to analyze the impact of *C*. *albicans* infection, both with and without *C*. *militaris* extract treatment, and at specific time intervals of 1, 24, and 48 h. *Galiomicin* gene expression is significantly influenced by treatment (two-way ANOVA: *p* < 0.0001), suggesting that treatment plays a primary role in enhancing gene expression. Time does not significantly affect *galiomicin* expression levels (*p* = 0.7761), and there is no interaction effect between treatment and time (*p* = 0.8386), indicating that the treatment effect is consistent across the time points.

**Figure 9:**
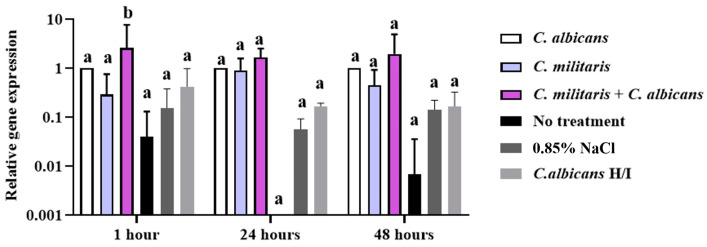
The relative gene expression of *galiomicin* in *G. mellonella* was observed by qRT-PCR to analyze the impact of *C. albicans* infection in *G. mellonella* after treatment with *C. militaris* extract at specific time intervals of 1, 24, and 48 h. ^a, b^ indicate a significant difference (*p* < 0.05) compared to the *C. albicans*-infected control group. *Galiomicin* gene expression is significantly influenced by treatment (two-way ANOVA: F(5, 36) = 15.60, dF = 5, *n* = 3, *p* < 0.0001). Time does not have a significant effect on gene expression levels (two-way ANOVA: F(2, 36) = 0.2552, dF = 2, *n* = 3, *p* = 0.7761), and there is no significant interaction between treatment and time (two-way ANOVA: F(10, 36) = 0.5551, dF = 10, *n* = 3, *p* = 0.8386).

Interestingly, the upregulation of the *galiomicin* gene was observed as early as 1 h after infection, showing a significant 2.57-fold increase in *G. mellonella* after infection with *C. albicans* and treated with *C. militaris* extract. Thus, 1 h after *G. mellonella* was infected, the maximal regulation of *galiomicin* gene expression was observed and followed by increases of 1.65-fold and 1.95-fold at 24 and 48 h, respectively. In contrast, the group treated with heat-inactivated (H/I) *C. albicans* had a reduced effect on *galiomicin* expression in *G. mellonella* compared to live *C. albicans*. There were no significant changes in *galiomicin* levels in the heat-inactivated group, similar to the observation in the no-treatment group. Additionally, when compared to 0.85% NaCl (Figure 9), the relative expression of the *galiomicin* gene was consistently high at 1, 24, and 48 h after infection with *C*. *albicans* and treated with *C*. *militaris* extract.

#### 3.7.2. Determination of *Gallerimycin* Gene Expression in the *G. mellonella* Model

**Figure 10:**
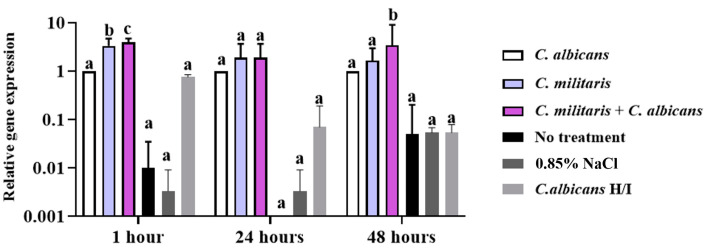
The relative gene expression of *gallerimycin* was observed by qRT-PCR to analyze the impact of *C*. *albicans* infection in *G. mellonella* treated with *C*. *militaris* extract at specific time intervals of 1, 24, and 48 h. ^a, b, c^ indicate a significant difference (*p* < 0.05) compared to the *C. albicans*-infected control group. *Gallerimycin* gene expression is significantly influenced by both treatment (two-way ANOVA: F(5, 36) = 39.55, dF = 5, *n* = 3, *p* < 0.0001) and time (two-way ANOVA: F(2, 36) = 5.813, dF = 2, *n* = 3, *p* = 0.0065). There is no significant interaction between treatment and time (two-way ANOVA: F(10, 36) = 2.093, dF = 10, *n* = 3, *p* = 0.0515).

The expression of the *gallerimycin* gene, a defensin-like peptide, is shown in Figure 10 at 1, 24, and 48 h after infection with *G. mellonella*. The relative expression of the *gallerimycin* gene in *G. mellonella* was upregulated by 100%, an early and significant event compared to the *C. albicans*-infected control group. The *gallerimycin* gene expression is significantly influenced by treatment (two-way ANOVA: *p* < 0.0001) and time (*p* = 0.0065), with no significant interaction between treatment and time (*p* = 0.0515), indicating a consistent effect of treatment across time points. Upregulation happened within 1 h in the groups that were treated with *C. militaris* extract, by 3.32-fold, as well as *C. albicans*-infected *G. mellonella* with *C. militaris* treatment, by 3.95-fold. However, the expression of *gallerimycin* at 24 h showed no significant difference between the groups treated with *C. militaris* extract and *C. albicans*-infected *G. mellonella* with *C. militaris* treatment, when compared to the *C. albicans*-infected control group. Additionally, the expression of *gallerimycin* at 48 h was significantly upregulated by 3.42-fold after infection with *C. albicans* and treated with *C. militaris* extract, and the expression was higher than the *C. albicans*-infected control group (Figure 10). In the group treated with heat-inactivated (H/I) *C. albicans*, there was a reduced impact on *gallerimycin* expression in *G. mellonella* compared to those infected with live *C. albicans*. Additionally, the levels of *gallerimycin* in the heat-inactivated group showed no significant differences, resembling the results that were seen in the no-treatment group.

#### 3.7.3. Determination of *Lysozyme* Gene Expression in the *G. mellonella* Model

**Figure 11:**
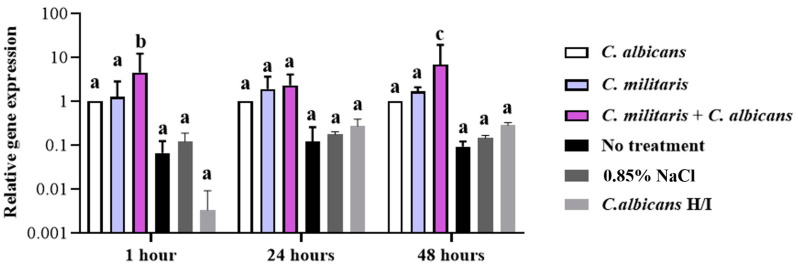
The relative gene expression of the *lysozyme* was observed by qRT-PCR to analyze the impact of *C*. *albicans* infection on *G. mellonella* treated with *C*. *militaris* extract at specific time intervals of 1, 24, and 48 h. ^a, b, c^ indicate a significant difference (*p* < 0.05) compared to the *C. albicans*-infected control group. *Lysozyme* gene expression is significantly influenced by treatment (two-way ANOVA: F(5, 36) = 13.24, dF = 5, *n* = 3, *p* < 0.0001), whereas time does not have a significant effect on expression levels (two-way ANOVA: F(2, 36) = 1.178, dF = 2, *n* = 3, *p* = 0.3194), and there is no significant interaction between treatment and time (two-way ANOVA: F(10, 36) = 1.314, dF = 10, *n* = 3, *p* = 0.2602).

The relative expression of the *lysozyme* gene in *G. mellonella* after *C. albicans* infection and treatment with *C. militaris* extract was significantly different compared to the *C. albicans*-infected control group, by 4.4-fold upregulation, when observed as early as 1 h, whereas the expression of the *lysozyme* gene at 24 h showed no significant difference between the groups treated with *C. militaris* extract, and *C. albicans*-infected *G. mellonella* with *C. militaris* treatment, compared to the *C. albicans*-infected control group. Moreover, the maximum regulation of the *lysozyme* gene was noted to show significant differences at 48 h within the same groups (Figure 11). The expression of the *lysozyme* gene in *G. mellonella* after *C. albicans* infection and treatment with *C. militaris* extract significantly increased by 6.8-fold. In the group treated with heat-inactivated (H/I) *C. albicans*, there was a diminished effect on *lysozyme* expression in *G. mellonella* when comparing to the group that was infected with live *C. albicans*. Furthermore, the *lysozyme* gene levels in the heat-inactivated group did not show significant changes, which was similar to the observations made in the no-treatment group. The treatment factor had a statistically significant impact on *lysozyme* expression (two-way ANOVA: *p* < 0.0001), while time as a factor did not significantly affect expression levels (*p* = 0.3194). Furthermore, no significant interaction was observed between treatment and time (*p* = 0.2602).

## 4. Discussion

To our knowledge, this study is the first to utilize the greater wax moth *G. mellonella* as a model host for investigating the gene expression of selected antimicrobial peptides in fat body and the number of hemocytes following *C. albicans* infection and treatment with *C. militaris* aqueous extract. Previous studies have reported the antibacterial, antioxidant, and anti-inflammatory properties of *C. militaris* extract, demonstrating its effectiveness against skin pathogenic bacteria such as *Staphylococcus aureus*, *Pseudomonas aeruginosa*, *Cutibacterium acnes,* and methicillin-resistant *S. aureus* (MRSA). Moreover, antioxidant activity was also observed from *C. militaris* extracts [44].

In this study, water was selected for extraction due to its polar protic nature, which effectively extracts polar components from medicinal herbs. The quality of the crude extract is affected by various factors, such as extraction methods, temperature, duration, and solvent polarity, ensuring nontoxicity and experimental integrity [45].

Moreover, this study has sparked curiosity in host–microbe interactions using insects as models [16]. The *G. mellonella* model has been successfully used for the study of *C. albicans* pathogenesis. Infected larvae displayed melanization, turning gray within an hour of infection, leading to complete melanization and mortality within 24 h. The melanization has also been described by Rueda et al. [46] during *Candida* infections; the phenoloxidase cascade occurs and results in melanization of the larvae. This response has been associated with β-glucans on the cell wall surface. Therefore, the degree of melanization of larvae indicates larval health.

The insects use a combination of cellular and humoral responses to defend themselves from invaders. The demarcation between cellular and humoral immune reactions as two distinct categories is not straightforward. This is because many humoral factors affect hemocyte functions, and hemocytes themselves are an important source of many humoral molecules. There is also a considerable overlap between cellular and humoral immune functions that span from recognition of foreign intruders to clot formation [47]. The humoral immune response involves the production of various antimicrobial peptides (AMPs) that can arrest and kill pathogens that evade the cellular immune response [48]. The AMPs are mostly produced in the fat body, hemocytes, the digestive system, salivary glands, and the reproductive tract of insects [10]. Thus, the infection model is correlated to both the hemolymph and hemocytes of infected larvae. During infection, there is an increase in hemocyte concentration along with the expression of antimicrobial peptide (AMP)-encoding genes [37]. Live *C. albicans* triggers immune activation that mitigates significant hemocyte loss and stimulates an increased production of hemocytes, leading to stable or even elevated hemocyte counts in response to the infection [49]. Similarly, treatment with *C. militaris*, known for its immunomodulatory properties, stimulates the innate immune system of *G. mellonella* [50]. This activation promotes the proliferation and functionality of hemocytes, which are essential for the defense mechanisms in insects. In contrast, the no-treatment group and heat-inactivated (H/I) *C. albicans* group exhibited a weaker immune response due to the absence of active pathogens. This reduced stimulation may result in increased apoptosis (programmed cell death) of hemocytes [47]. Consequently, in the absence of such immune activation, hemocyte levels decline in these groups.

The hemolymph of *G. mellonella* contains immune-relevant proteins including lysozymes, which support cellular and humoral responses and influence host–parasite interactions [51]. Antimicrobial activity was proven by treating *G. mellonella* larvae with *C. militaris* extract, which increased the survival of *G. mellonella* larvae against *C. albicans* infection, where substantial antimicrobial activity was detected in the larval hemolymph. Cordycepin also reduced the mortality rate of larvae infected with *C. albicans* until the end of the experiment by 72 h. Therefore, cordycepin is recommended to counter unfavorable conditions caused by the host immune system [52]. The transcription of AMP-related genes in response to bacterial lipopolysaccharides has revealed proteins that enhance immunity [53]. This study opens possibilities to investigate changes in gene-encoded antimicrobial peptides that are expressed both before and after treatment. Transcriptional expression profiles of AMP-related genes have been widely used to study the capabilities of larval immune systems that express antimicrobial peptides in response to microorganisms. The best characterized *G. mellonella* AMPs are *gallerimycin*, *galiomicin*, *cecropins*, *cobatoxin*, *moricins*, and *gloverins*, of which *gallerimycin* and *galiomycin* are the most studied AMPs [37]. After *C. albicans* infection, the expression of antimicrobial peptide (AMP) genes, such as *galiomicin*, *gallerimycin*, and *lysozyme*, was induced. Recently, a study found that cordycepin, a secondary metabolite of *C. militaris*, can act as an insect immune modulator [54]. However, a high dose of cordycepin can decrease the regulation of antimicrobial peptide genes in the immune system response to fungal infection, resulting in rapid death of the insect host [54].

*G. mellonella* was injected into the hemocoel in this study, and the AMP genes were expressed in the fat body. Crude extract of *C. militaris* at nontoxic doses could stimulate an immune response and suddenly upregulate antimicrobial peptide genes that can be observed as early as 1 h after infection. Furthermore, Bergin et al. [55] reported similar findings, with increased expression of *gallerimycin* and *galiomicin* between 8 and 24 h after exposure to sublethal doses of *C. albicans*. These findings aligned with our research, which also showed an early increase in the expression of genes responsible for the production of *gallerimycin* and *galiomicin* in *G. mellonella* after infection with *C. albicans* and treatment with *C. militaris*. Furthermore, our study revealed that the group treated with heat-inactivated (H/I) *C. albicans* exhibited a diminished effect on the expression of antimicrobial peptide (AMP) genes in *G. mellonella* compared to those infected with live *C. albicans*. Previous research indicates that the immune response, including AMP production, is significantly reduced when exposed to heat-inactivated pathogens compared to live infections [56]. This suggests that live *C. albicans* induce a more robust immune activation compared to its heat-inactivated form [57].

Previous studies have documented that *gallerimycin* and *galiomicin* are antifungal defensins that synthesize via the Toll receptor (TLR) pathway in response to entomopathogenic fungi infections [58,59]. Lysozyme has both antibacterial and antifungal activity [60]. However, the important aspects of the humoral response, such as clotting, ROS generation, and primary immunization of *G. mellonella* larvae in response to *C. albicans* infection and treatment with *C. militaris* extract, have not been studied. These factors are important for a better understanding of *G. mellonella* as a model organism for studying host–pathogen interactions and infection treatments.

It is possible that the extracts from *C. militaris* and other entomopathogenic fungi, such as *Beauveria bassiana*, influence the immune system of *G. mellonella*, as these extracts could upregulate the expression of important antimicrobial peptide genes [54]. Interestingly, in a previous study, the AMP and *galiomicin* genes were not upregulated when *C. militaris* fungi were infected in *G. mellonella*. It might be due to the route of infection, and the expression of the AMP gene may be found throughout the insect tissue rather than just in the fat body [59]. In our study, however, *C. militaris* extract enhanced the immune response during *C. albicans* infection of *G. mellonella* by recruiting hemocytes and increasing the expression of antimicrobial peptides, including *galiomicin*, *gallerimycin*, and *lysozyme*. These peptides play a crucial role in pathogen defense by disrupting the integrity of the fungal cell wall or membrane [61], similar to other antimicrobial mechanisms observed in invertebrate immune systems. Additionally, the heightened melanization response could encapsulate and neutralize *C. albicans* cells, preventing further proliferation [62]. Compounds in *C. militaris*, such as cordycepin, possess antimicrobial, antioxidant, and immune-modulating properties. These compounds potentially enhance pathogen clearance by stimulating phagocytosis and boosting hemocyte activity [63]. This cascade of immune responses strengthens the host’s ability to combat fungal infections. While more research is needed to clarify the precise molecular targets of *C. militaris* extract within *C. albicans*, this proposed mechanism underscores the multifaceted role of the extract in immune stimulation.

To date, most research on cordycepin has focused on innate mammalian responses rather than the insect immune system [64,65]. Similarities exist between mammalian and insect immunity, including the homology between the Toll-like receptor in mammals and the Toll receptor in insects, as well as phagocytosis and clotting cascades against invasive pathogens [14]. Therefore, our research may support the use of *C. militaris* extract for immunomodulation in the *G. mellonella* model, which may impact treatment of the insects and mammals during *C. albicans* infections.

## 5. Conclusions

In this study, the immune response of *G. mellonella* larvae was examined before and after treatment with *C. militaris* extract in the context of *C. albicans* infection. We found that *C. militaris* significantly enhanced the immune response of *G. mellonella*, as demonstrated by increased hemocyte recruitment and upregulation of antimicrobial peptide gene expression, including *galiomicin*, *gallerimycin*, and *lysozyme*. These genes were upregulated to maximum levels of expression within 1 h, suggesting rapid immunomodulation. However, while these findings demonstrate the potential of *C. militaris* extract to modulate the immune system and offer protection against *C. albicans*, the inherent differences between insect and mammalian immune systems pose challenges when translating these results to human applications. Despite these limitations for the use of *G. mellonella,* as the larvae lack organs and an adaptive immune system, the use of the larvae is convenient and cost effective with many applications. Further studies are needed to fully elucidate the effects of *C. militaris* extract in more complex immune systems and to assess long-term implications. Therefore, the *G. mellonella* model provides valuable insights into potential therapies for *C. albicans* infections and serves as a foundation for future research.

## Figures and Tables

**Table 1:** Antimicrobial peptide primers used for quantitative reverse transcription polymerase chain reaction (qRT-PCR).

Genes	The Sequence of PCR Primers (5′–3′)	References
*galiomicin*	F: CCTCTGATTGCAATGCTGAGTG R: GCTGCCAAGTTAGTCAACAGG	[42]
*gallerimycin*	F: GAAGATCGCTTTCATAGTCGC R: TACTCCTGCAGTTAGCAATGC	[42]
*lysozyme*	F: GGACTGGTCCGAGCACTTAG R: CGCATTTAGAGGCAACCGTG	[43]
*β-actin*	F: GGGACGATATGGAGAAGATCTG R: CACGCTCTGTGAgvGGATCTTC	[42]

**Table 2:** The *G. mellonella* health index score.

Day	Conditions					
	No Treatment	0.85% NaCl	10^6^ H/I	10^6^	10^5^	10^4^
0	Injection					
1	1	1	1	0	1	1
2	1	1	1	0	1	1
3	1	0.5	0.5	-	1	1
4	1	0.5	0.5	-	1	1
5	0.5	0.5	0.5	-	0.5	0.5
6	0.5	0.5	0.5	-	0.5	0.5
7	0.5	0.5	0.5	-	0.5	0.5

Healthy index is indicated as unhealthy (0), partial healthy (0.5), healthy (1), and dead larvae (-).

## Data Availability

The original contributions presented in the study are included in the article/Supplementary Material; further inquiries can be directed to the corresponding author.

## Supplementary Materials

The following supporting information can be downloaded at: https://www.mdpi.com/article/10.3390/insects15110882/s1, Table S1: Weight of *G. mellonella* larvae in toxicity test of *C. militaris* extract; Table S2: Survival of *G. mellonella* larvae after treatment with *C. militaris* extract; Table S3: Weight of *G. mellonella* larvae in *C. albicans* infection; Table S4: Survival of *G. mellonella* larvae after *C. albicans* infection; Table S5: Weight of *G. mellonella* larvae used in the infection of *C. albicans* at 5 × 10^5^ cells/larvae and treated with *C. militaris* extract at 0.125 and 0.25 mg/larvae; Table S6: Survival of of *G. mellonella* larvae after infection with *C. albicans* at 5 × 10^5^ cells/larvae and treated with *C. militaris* extract at 0.125 and 0.25 mg/larvae; Table S7: The hemocytes of *G. mellonella* after *C. albicans* infection at 5 × 10^5^ cells/larvae and treated with *C. militaris* extract at 0.125 mg/larvae for 1, 24, and 48 h.
